# Fabrication of 3D Functional Nanocomposites Through Post‐Doping of Two‐Photon Microprinted Nanoporous Architectures

**DOI:** 10.1002/smll.202403405

**Published:** 2024-12-17

**Authors:** Junning Zhang, Sida Liu, Kannasoot Kanokkanchana, Mariia Kuzina, Meijun Zhou, Xin Du, Zhongze Gu, Zheqin Dong, Pavel A. Levkin

**Affiliations:** ^1^ Institute of Biological and Chemical Systems–Functional Molecular Systems (IBCS‐FMS) Karlsruhe Institute of Technology (KIT) Hermann‐von‐Helmholtz‐Platz 1 76344 Eggenstein‐Leopoldshafen Germany; ^2^ State Key Laboratory of Digital Medical Engineering School of Biological Science and Medical Engineering Southeast University Nanjing 211189 China; ^3^ Technical University of Munich Campus Straubing for Biotechnology and Sustainability Uferstraße 53 94315 Straubing Germany; ^4^ Department of Additive Manufacturing, School and Hospital of Stomatology Cheeloo College of Medicine, Shandong University & Shandong Key Laboratory of Oral Tissue Regeneration & Shandong Engineering Research Center of Dental Materials and Oral Tissue Regeneration & Shandong Provincial Clinical Research Center for Oral Diseases No. 44‐1 Wenhuaxi Road Jinan Shandong 250012 China; ^5^ Institute of Organic Chemistry (IOC) Karlsruhe Institute of Technology (KIT) Kaiserstraße 12 76131 Karlsruhe Germany

**Keywords:** 3D printing, liquid repellent, nanoporous, surface wettability, two‐photon lithography

## Abstract

Two‐photon lithography (TPL) enables the fabrication of complex 3D structures with sub‐micrometer precision. Incorporation of new functionalities into TPL‐printed structures is key to advance their applications. A prevalent approach to achieve this is by directly adding functional nanomaterials into the photoresist (called “pre‐doping”), which has several inherent challenges including material compatibility, light scattering, and nanoparticle agglomeration. Here, a conceptually different “post‐doping” strategy is proposed, where the functionality of the TPL‐printed architectures is achieved by impregnating functional materials into their nanoporous 3D mimics. Using the principle of polymerization‐induced phase separation, TPL printing of complex microarchitectures with well‐defined nanoporous structures having pores of ≈420 nm is realized, which allows spontaneous impregnation of functional liquids via capillary effect. Importantly, unlike the “pre‐doping” approach that requires printing optimization for each photoresist, this strategy is highly versatile in terms of functionalities possible. As a proof‐of‐concept, the impregnation of several functional liquids into TPL‐printed porous microstructures is demonstrated: a fluorinated‐lubricant, an ionic liquid, and three types of fluorescent liquids, conferring the microstructures with slippery, conductive, and localized fluorescence properties, respectively. Such versatility to fabricate complex microstructures with tailorable and localized functionalities is expected to open new possibilities in wide fields including bionics, electronics, and cell biology.

## Graphical abstract

A “post‐doping” strategy is proposed for creating versatile functional microstructures by impregnating functional materials into two‐photon lithography‐printed porous structures. This approach enables the simple fabrication of microstructures with conductive, low surface adhesion, and fluorescent properties. It overcomes the limitations of pre‐doping, such as limited material compatibility and interference with photopolymerization, expanding the functional potential of TPL‐printed architectures.

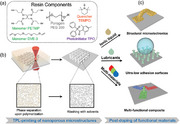

## 1 Introduction

Over the past few years, TPL has gained significant attention as a promising technique for fabricating intricate, small‐scale 3D architectures.^[^ ^1^, ^2^, ^3^, ^4^ ^]^ TPL employs a femtosecond laser focused on a negative photoresist to initiate localized polymerization. By rastering the laser in 3D, microstructures with resolutions down to tens of nanometers and virtually any geometry can be created. This method's flexibility paves the way for numerous technological applications, encompassing bionics,^[^ ^5^ ^]^ electronics,^[^ ^6^ ^]^ drug delivery,^[^ ^7^ ^]^ analysis,^[^ ^8^ ^]^ and basic cell biology^[^ ^9^ ^]^ study.

To widen the applicability of these 3D structures, it is essential to not only meticulously engineer their architecture but also modulate their functionalities.^[^ ^10^, ^11^, ^12^, ^13^ ^]^ One common approach, termed “pre‐doping”,^[^ ^14^ ^]^ involves incorporating functional components, such as nanoparticles,^[^ ^15^ ^]^ graphene,^[^ ^16^ ^]^ or other materials^[^ ^17^, ^18^, ^19^, ^20^, ^21^, ^22^ ^]^ into the photoresist, which is subsequently integrated into the structure during photopolymerization. This method achieves the fabrication of 3D structures with magnetic,^[^ ^23^ ^]^ luminescent,^[^ ^24^ ^]^ and electrical properties.^[^ ^6^ ^]^ However, “pre‐doping” methods have some inherent challenges. In most cases, functional components are neither polymerizable nor easily soluble. Introducing them usually decreases the photoresist's processability.^[^ ^8^ ^]^ In addition, opaque functional components, such as nanoparticles, hinder the passage of laser light through the photoresist, limiting photopolymerization. These challenges restrict both the variety and concentration of dopants that can be used, thereby further restricting the functionality and performance of the resultant microstructures. Due to the inherent drawbacks of pre‐doping methods, post‐functionalization of microstructures—such as surface chemical modification or physical vapor deposition—has emerged as a promising complementary approach. However, these methods are limited to grafting chemical groups or depositing a small selection of metals or semiconductor materials, which restricts their applicability. A more detailed comparison can be found in the Supporting Information.

**Figure 1:**
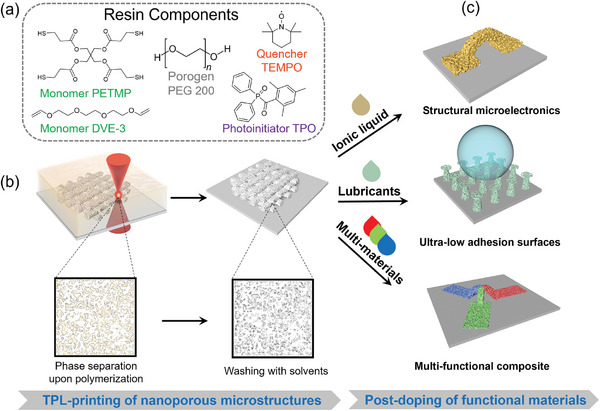
Concept and mechanism of functionalization of a porous microstructure platform with post‐doping. a) Components of the photoresist for TPL printing of porous microstructures. b) The resist polymerizes upon laser scanning, causing simultaneous phase separation and resulting in a polymer‐rich phase and a porogen‐rich phase. The porogens are subsequently removed to afford the porous structure. c) The pores of the TPL‐printed microarchitectures can be filled with various materials to obtain the desired functionality.

Here, we present a novel method for constructing composite functional microstructures based on a “post‐doping” strategy (**Figure** **1**). Utilizing the principle of polymerization‐induced phase separation,^[^ ^25^, ^26^ ^]^ we successfully demonstrated TPL printing of intricate nanoporous microarchitectures with pore sizes ≈420 nm. Subsequently, these porous microstructures can be impregnated with liquid functional components, which spontaneously penetrate the pores through capillary action. Alternatively, the microstructures can be immersed in nanoparticle dispersions, allowing the pores to capture functional nanoparticles. In essence, the porous microstructure serves as a matrix for accommodating the functional components. Crucially, our “post‐doping” approach, in contrast to the “pre‐doping” method, does not require individual printing optimization for each photoresist. Therefore, our method offers significant versatility by being largely independent of the specific functional components employed. As a proof‐of‐concept, we infused the TPL‐printed microstructures with several distinct functional fluids: a fluorinated lubricant, an ionic liquid, and fluorescent liquids, each imparting their respective properties. This endowed the microstructures with slippery, ionically conductive, and fluorescent properties, respectively. We also infused multiple functional components into a single microstructure, allowing for combined functionality and broadening the application potential of the “post‐doping” method. We anticipate this new concept for the fabrication of functional 3D microstructures will find applications in fields like bionics, electronics, and drug delivery.

## 2 Results and Discussion

To fabricate 3D microarchitectures with inherent porosity, we combined TPL with the principle of polymerization‐induced phase separation (Figure S1, Supporting Information). The specific photoresist consisted of monomers, porogen, photoinitiator, and quencher (Figure 1a). Pentaerythritol tetrakis(3‐mercaptopropionate) (PETMP) and tri(ethylene glycol) divinyl ether (DVE‐3) were selected as the monomers for their rapid polymerization ability via thiol‐ene click chemistry.^[^ ^27^ ^]^ Compared to acrylate polymerization,^[^ ^28^ ^]^ this system is insensitive to oxygen inhibition, eliminating the need for intricate oxygen‐removal procedures.^[^ ^29^, ^30^ ^]^ polyethylene glycol 200 (PEG 200) was selected as the porogen owing to its excellent miscibility with the monomers but immiscibility with the polymers, as well as its low evaporation rate and viscosity favorable for printing. Diphenyl(2,4,6‐trimethylbenzoyl)phosphine oxide (TPO) was selected as the photoinitiator based on its absorption spectrum matching the emission spectrum of the TPL printer used. The quencher, 2,2,6,6‐tetramethylpiperidinyloxyl (TEMPO), was introduced into resist to inhibit rapid self‐polymerization. Laser scanning triggered the polymerization of monomers, causing simultaneous phase separating and resulting in the formation of a polymer‐rich and a polymer‐poor phase. This photoresist can be processed at a laser scanning speed of 1–10 cm s^−1^, which is comparable with conventional acrylate resins, such as the IP series photoresist. However, for functionalized acrylate resins that have undergone “pre‐doping” treatments, the processing speed usually decreases due to interference from the dopants, dropping to a range of 0.05–1 cm s^−1^,^[^ ^16^ ^]^ or even lower.^[^ ^6^, ^31^ ^]^ At this point, the advantage of our method in terms of microstructure fabrication speed becomes more apparent. After the printing was completed, the architectures were washed with solvent followed by critical point drying (CPD), which left behind porous structures within the 3D microarchitectures. We mixed the two monomers, PETMP and DVE‐3, in molar ratios of 1:2 and 1.5:2, allowing the thiol and vinyl groups to react at molar ratios of 1:1 and 1.5:1, respectively. After polymerization, the materials were characterized using Raman spectroscopy (Figure S2, Supporting Information). It was observed that when the molar ratio of [SH] to [C═C] was 1.5:1, a characteristic peak for thiol appeared at 2574 cm^⁻¹^, indicating the presence of unreacted thiol groups in the polymer. However, when the molar ratio of [SH] to [C═C] was 1:1, the thiol signal completely disappeared, suggesting that the thiol groups had been fully consumed, due to the reaction between the thiol and vinyl groups, thereby confirming that polymerization occurred between PETMP and DVE‐3.

**Figure 2:**
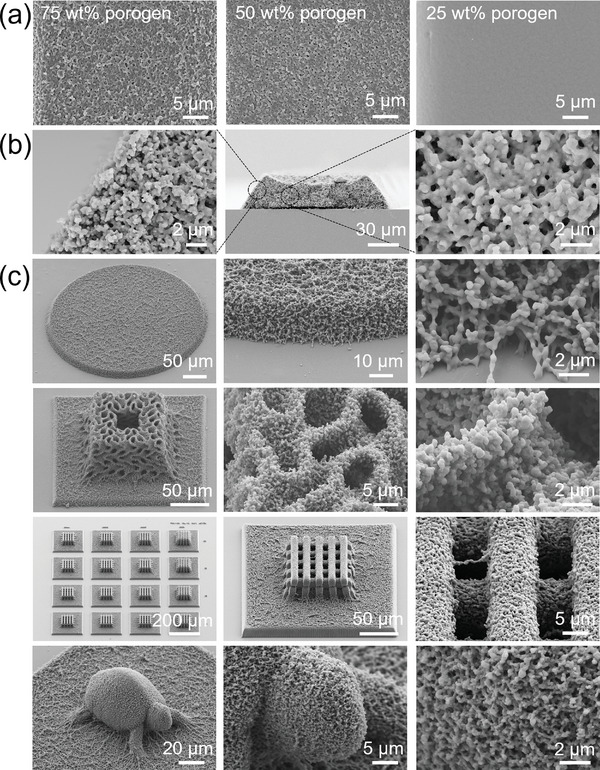
SEM images of the porous microstructures. a) Top view SEM images of porous microstructures with different porogen concentrations. b) Cross‐sectional SEM images of the porous microstructure, showing from left to right the edge, overall view, and middle region of the cross‐section. c) SEM images of 3D porous microstructures at different magnifications.

To explore the impact of porogen content on the porous structures of the 3D‐printed microarchitectures, a systematic investigation was conducted. The results showed that the porosity of the 3D microarchitectures increased with higher porogen content (**Figure** **2**a). At a porogen content of only 25 wt.%, the printed structure appeared dense without any visible pores. However, as the porogen content increased to 50 and 75 wt.%, porous structures were distinctly visible within the printed materials, leading to final porosities of 47% and 59%, respectively. The content of the porogen not only affects the porosities but also the pore distribution. When the porogen concentration was 50 wt.%, we observed non‐uniform pore distribution in the lower‐height microstructures, with the outer regions of the microstructures exhibiting low porosity or even being completely non‐porous (Figure S5, Supporting Information). We believe this phenomenon is caused by the polymerization process in these regions being able to draw more cross‐linker from the surrounding solution. When the porogen concentration was increased to 75 wt.%, this non‐uniform distribution was significantly improved. However, this improvement was accompanied by a notable increase in structural shrinkage, which led us to hypothesize that further increasing the porogen concentration would likely compromise the structural integrity, and therefore, higher concentrations were not tested. The cross‐sectional analysis of the printed microstructure revealed highly porous morphology throughout the entire architecture (Figure 2b), with an average pore diameter of ≈420 nm. Porosity and pore size distribution were obtained from the analysis of SEM images using imageJ (Figure S6, Supporting Information). Such a bulk nanoporous structure is crucial for ensuring the complete impregnation of functional liquids into the 3D‐printed microarchitectures. It is worth noting that CPD is a critical step to prevent pores from collapsing during the process (Figure S3, Supporting Information).

**Figure 3:**
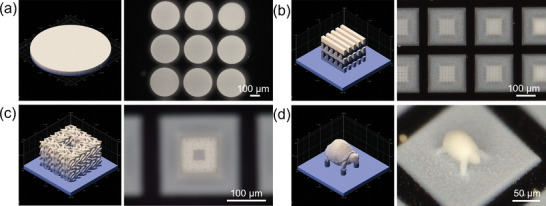
a–d) 3D model renderings and optical microscope images of the porous microstructures shown in Figure 2d.

To showcase the design capabilities of our approach, we printed several intricate 3D microarchitectures, including scaffolds, minimal surface structures, and turtle‐like structures (Figure 2c). **Figure** **3** shows the optical microscope images of the porous microstructures from Figure 2c.

To demonstrate our method's potential in creating 3D functional microstructures via post‐doping, we first infused the TPL‐printed porous architectures with a slippery lubricant, achieving ultra‐low liquid adhesion properties.

**Figure 4:**
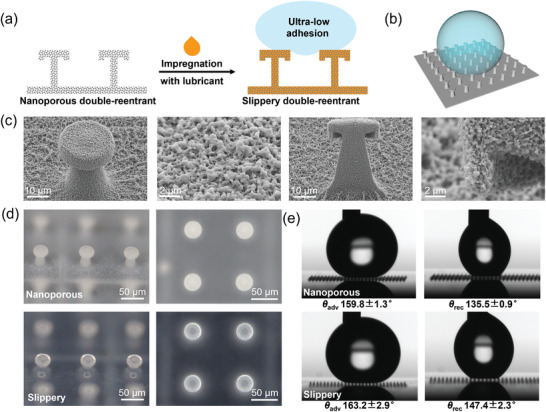
Preparation and application of ultra‐low adhesion microstructures based on porous materials. a,b) Schematic presentation of the fabrication of slippery double‐reentrant porous micropillars. c) SEM images of porous micropillars with surface and cross‐sectional views. d) Optical pictures of porous micropillars with 45° and top views. e) Advancing (*θ*_adv_) and receding (*θ*_rec_) contact angles of water on porous micropillars before and after lubrication.

Despite the successful utilization of TPL in creating double‐reentrant structures featuring excellent liquid repellency, these superomniphobic microstructures still exhibit considerable adhesion due to their substantial solid‐liquid contact area. In previous studies,^[^ ^32^ ^]^ we have employed a robotic liquid dispenser to apply a slippery lubricant layer on top of the double‐reentrant structures, which effectively reduces liquid adhesion. However, the fabrication process was complex and time‐consuming. Here, we show that the unique nanoporosity of double‐reentrant structures allows for the spontaneous infiltration of the slippery lubricant through the capillary effect, thus greatly simplifying the fabrication process (**Figure** **4**a).

In order to minimize liquid contact, we arranged the porous double reentrant pillars with a diameter (D) of 33 µm in a square array (3 mm × 3 mm) with a spacing (P) of 100 µm (Figure 4b). SEM results depicted the sharp overhanging geometry of the micropillars, a key characteristic for achieving superomniphobicity (Figure 4c). Moreover, the nanoporous structure was uniformly distributed throughout the entire micropillar, ensuring the complete impregnation of the lubricant. Krytox was selected as the lubricant due to its immiscibility with water, making it commonly used in supporting slippery liquid‐infused porous surfaces. According to the literature, Krytox demonstrates long‐term stability in hydrophobic applications, with durations ranging from 45 to 93 days,^[^ ^33^, ^34^ ^]^ making it more favorable for long‐term applications. As‐printed nanoporous micropillars appeared white due to strong light scattering. After lubricant impregnation, the micropillars maintained their shape but became more transparent, as the refractive‐index contrast between the lubricant and the polymer decreased compared to air (Figure 4d). As expected, the nanoporous micropillars exhibited repellency toward water (Figure 4e), yet with substantial adhesion as evident by the large contact angle hysteresis values observed (24.3° with receding water contact angles (WCA) of 135.5 ± 0.9°). However, after lubricant infusion, the contact angle hysteresis was significantly reduced (15.8° with receding WCA of 147.4 ± 2.3°), demonstrating a substantial reduction in adhesion at the lubricant‐liquid interface compared to the solid‐liquid interface. It is worth mentioning that such a slippery Cassie state is applicable only when the size of liquid droplets considerably exceeds that of the micropillars. When the droplet size becomes smaller than the spacing between the micropillars, the liquid droplet becomes trapped, and the slippery Cassie state no longer exists. However, the slipperiness of these micropillars may still reduce droplet adhesion, which requires further investigation. We anticipate this straightforward method for fabricating low‐adhesion super‐repellent surfaces to hold promise for various applications including anti‐biofouling, anti‐icing, and zero‐loss liquid transportation.

Recently, the development of conductive 3D microstructures has garnered attention due to their promising roles in microelectronics and burgeoning areas like flexible electronics,^[^ ^35^ ^]^ nanophotonics,^[^ ^36^ ^]^ and plasmonics.^[^ ^37^ ^]^ While TPL offers high machining accuracy, making it a favored method for creating 3D microstructures, producing conductive variants of these structures still poses considerable challenges.

**Figure 5:**
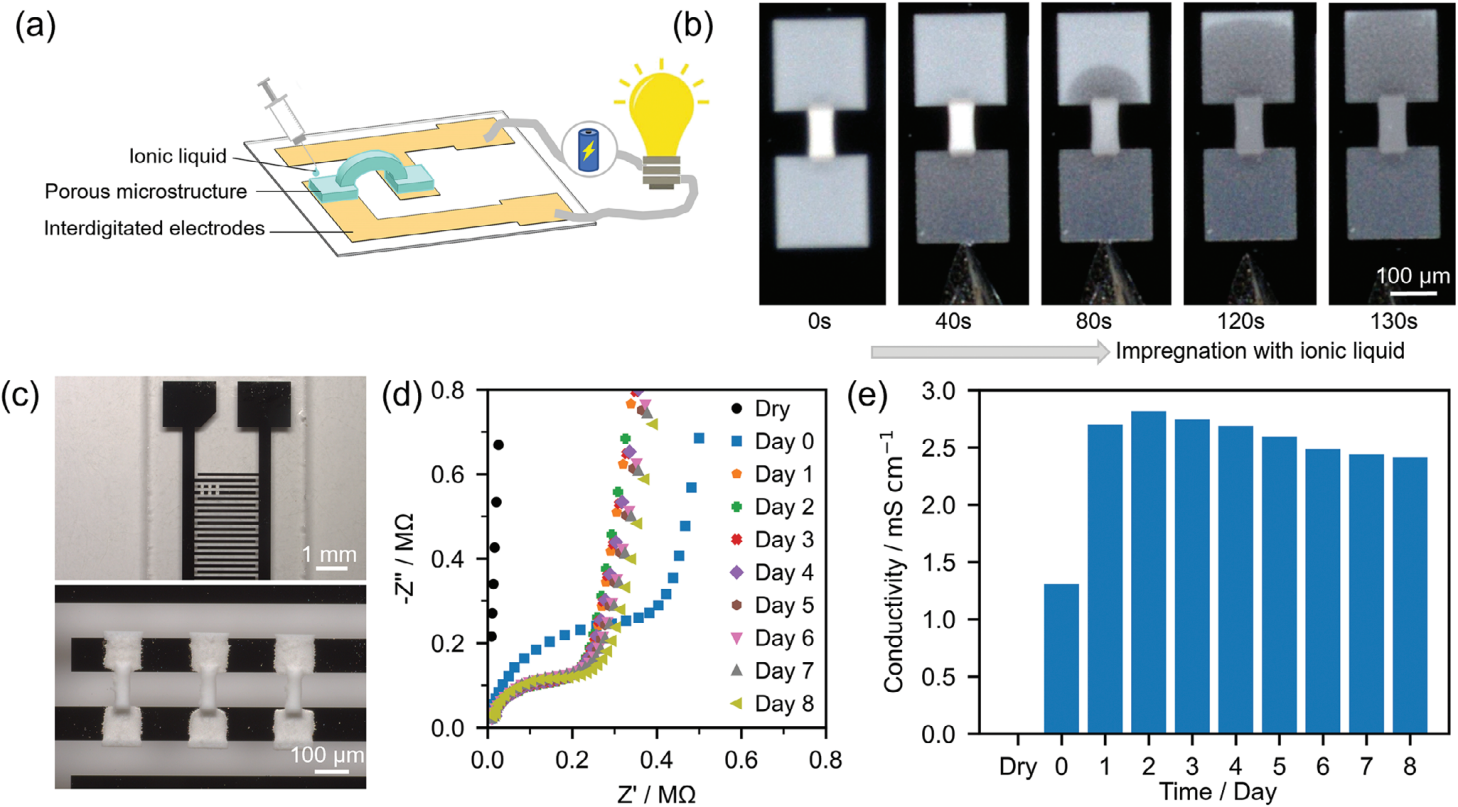
Preparation and application of conductive microstructures based on porous materials. a) Schematic representation of the fabrication of conductive, ionic liquid‐filled, porous bridges‐like microstructures. b) Process of filling porous microstructures with ionic liquids. c) Optical image of porous bridge‐like microstructures that are 3D printed between the prongs of interdigitated electrodes. d) The EIS of the porous microstructures filled with ionic liquids e) The changes in ionic conductivity over time for the porous microstructures filled with ionic liquids.

We demonstrate the potential functionality of TPL‐printed porous microstructures as conductive structures, with a notable enhancement achievable by incorporating room‐temperature ionic liquids (RTIL), as depicted in **Figure** **5**a. The spontaneous absorption of RTIL triethylsulfonium bis(trifluoromethylsulfonyl)imide([SEt3]NTf2) is illustrated through time‐lapse images in Figure 5b and Video S1 (Supporting Information); within a span of 130 s, the RTIL uniformly permeates the entire length (500 µm) of the microstructure. To investigate the effect of introducing the RTIL, electrochemical measurements were conducted on the bridge microstructures that were printed on the interdigitated electrodes (IDE, Figure 5c). Cyclic voltammograms (Figure S7, Supporting Information) reveal no discernible differences between dry structures and those infused with RTIL, indicating the electrochemical stability of RTIL within the porous matrix. The conductivity of the RTIL‐infused bridge sample, measured by electrochemical impedance spectroscopy (EIS, see the Nyquist plot in Figure 5d), gradually increases from zero to a maximum value of 2.7 mS cm^−1^ after two days (Figure 5e). The increase in conductivity might be attributed to the slow diffusion of the ionic liquid into the nanoporous structures. Meanwhile, over time, we observed a decrease in the conductivity of the conductive microstructures, which became noticeable starting from the third day. Therefore, we focused on the long‐term stability of this conductive microstructure. After 6 months, the conductivity of the original sample decreased to 45% of its peak value on the 2nd day, while the double‐layer capacitance increased 3.1 times during the same period, likely due to the degradation of the ionic liquid and moisture absorption (Figure S8a, Supporting Information). A newly prepared sample, infused with the same 6‐month‐old ionic liquid, showed similar conductivity and capacitance as the aged original sample, even from the first day of measurement (Figure S8b, Supporting Information). It indicates that the conductivity decrease is primarily due to the change in the ionic liquid's properties over time. In comparison with other studies on conductive microstructures, the conductivity is highly dependent on the type of dopant. When metal compounds are used as dopants, conductivity can reach values of 1040–1060 mS cm^−1^.^[^ ^3^ ^]^ When the dopants are carbon nanotubes or Poly(3,4‐ethylenedioxythiophene) polystyrene sulfonate(PEDOT), the conductivity typically ranges from 10 to 500 mS cm^−1^.^[^ ^3^ ^]^ For ionic liquids, the conductivity can reach 1–10 mS cm^−1^,^[^ ^38^ ^]^ which is comparable to our material. It is worth noting that in our validation experiments, the porous microstructures infused with ionic liquids exhibited a composite conductivity of 2.7 mS cm^−1^, though this is not the upper limit of what can be achieved. If saturated sodium chloride solutions were infused, it is expected that the conductivity could be increased to ≈250 mS cm^−1^.^[^ ^39^ ^]^ In summary, despite certain limitations, the infusion of ionic liquids into porous microstructures crafted via TPL enables the fabrication of flexible, yet time‐efficient microstructures with enhanced electrical conductivity.

It is important to note that the porosity of the 3D microarchitectures not only serves the purpose of accommodating functional liquids but also enables the adsorption of functional nanoparticles. To prove this, we immersed the porous microstructure into a dispersion of 10 nm ferric oxide nanoparticles (17.7 wt.% in light hydrocarbon oil), followed by drying to remove the liquid. As a result, the white porous microstructure turned tan due to the infiltration of nanoparticles, as shown in Figure S9 (Supporting Information). The EDX results confirmed iron's distribution throughout the microstructure, including its center, thereby confirming that nanoparticles permeated the entirety of the 3D microstructure. Unlike the pre‐doping method, which requires compatibility testing and printing optimization for each functional nanoparticle, our approach is largely independent of the specific materials used, as long as the nanoparticles can be effectively dispersed in a liquid medium. This greatly broadens the range of potential functionalization options. We anticipate that more functional materials, such as other nanoparticles, polymers, and bioactive compounds, can be infused into the porous microstructures, enabling the construction of a wider variety of functional microstructures.

Adjusting the local functional properties within microscale materials with 3D complex shapes is crucial for developing sophisticated functionalities.^[^ ^40^, ^41^ ^]^ Here, we use the porous microstructures as a matrix for localized functional modifications. Since fluid diffusion in porous materials is confined by the capillary effect, injecting suitable fluids at specific locations allows for the localized functional editing of these materials.

**Figure 6:**
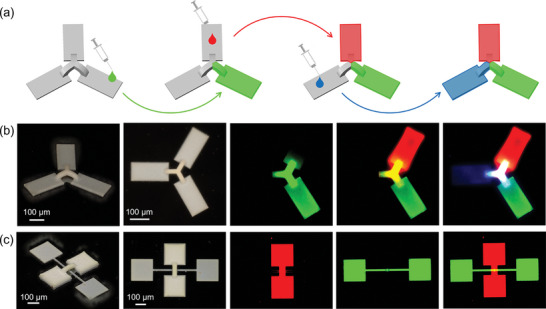
Preparation of multiple fluorescent microstructures based on porous materials. a) Schematic presentation of the fabrication of porous microstructures with multiple fluorescent regions. b) Optical and fluorescent images of clover‐leaf‐like porous microstructures, sequentially filled with green, red, and blue fluorescent liquids. c) Optical and fluorescent pictures of overpass‐like porous microstructures.

As a demonstration, we sequentially filled various fluorescent liquids into the TPL‐printed porous microstructures. For the interlinked geometry such as a clover‐leaf‐like structure, we sequentially filled red, green, and blue fluorescent liquids in the corresponding leaf part of the porous microstructure (**Figure** **6**a). The results show that the three fluorescent colors are independent of each other in the leaf part. In the petioles part, however, combined colors such as orange or white are visible due to the combination of different fluorescent liquids (Figure 6b). For the disjointed geometry such as overpass‐like structures. We filled two parts of the “overpass” with red and green fluorescent liquids, respectively. Since the porous microstructures are disjointed, the filled fluorescent liquids do not interfere with each other (Figure 6c). The process of filling the porous microstructure with fluorescent liquid is shown in Video S2 (Supporting Information). We expect that fluorescent liquids can be replaced by more targeted functional components, thereby demonstrating the possibility of preparing more complex microstructures with multiple functionalities. The uniform distribution of functional dopants within the porous microstructures is also an important aspect to investigate. We specifically infused a fluorescent liquid into one side of the porous microstructure. If non‐uniform distribution occurred during impregnation due to chromatographic effects, we would expect to observe a fluorescence intensity gradient from the source to the endpoint. However, confocal microscopy observations (Figure S10, Supporting Information) revealed a uniform fluorescence pattern, demonstrating that the post‐doping method is capable of achieving an uniform distribution of functional dopants.

## 3 Conclusion

In conclusion, we have developed an efficient method for constructing functional microstructures by impregnating functional components into TPL‐printed microarchitectures with inherent porosity. The unique bulk porosity of these structures acts as a matrix for absorbing and retaining specific components, thereby imparting corresponding functionalities to the 3D microstructures. We demonstrate that this technique is compatible with both functional liquid and solid nanoparticle materials, and offers flexibility in tailoring local properties. Significantly, unlike the common pre‐doping approach, which necessitates printing optimization for each functional ink, our approach is highly versatile in terms of functional materials. To showcase such versatility, we have successfully fabricated various functional microstructures such as a low‐adhesion double‐reentrant microstructure, a conductive micro‐bridge, and a leaf‐like microstructure with localized fluorescence. We believe our method introduces a new material concept for manufacturing 3D microstructures with complex shapes and sophisticated functionalities, opening up exciting possibilities in fields including micro‐electronics, micro‐robotics, and cell biology.

## 4 Experimental Section

### Chemicals

PETMP (95%), DVE‐3 (98%), TPO (97%), and TEMPO (98%) were obtained from Sigma–Aldrich; Krytox GPL103 Oil was used as the lubricant. PEG 200 and [SEt3]NTf2 (98%) were obtained from Alfa Aesar. The fluorescent liquids, primarily composed of hydrogen peroxide, phenyl oxalate ester compounds, and fluorescent dyes corresponding to the desired colors, were derived from commercially available glow sticks.

### Protocols—TPL Fabrication of 3D Porous Microstructures

The 3D printing process was carried out with a commercial device, the Photonic Professional GT from Nanoscribe GmbH, using a 25×/NA 0.8 objective lens–the Zeiss LCI Plan‐Neofluar 25×/0.8, in dip‐in mode. The structures depicted were fabricated using a focal velocity ranging from 1 to 10 cm s^−1^, a slicing distance of 1 µm, and a hatching distance of 0.5 µm. The laser power was set within a range of 100–40, corresponding to an actual output power of 50 to 20 mW, with a laser wavelength of 780 nm. For instance, the turtle‐like microstructure shown in Figure 2c was created using a laser power setting of 60 (≈30 mW) and a focal velocity of 5 cm s^−1^.

### Critical Point Drying

Following the 3D printing, the samples were submerged in acetone for 30 min. The next step involved CPD, executed using a commercially available device, the Leica EM CPD300. In this stage, the sample was moved into the equipment's chamber and chilled to 10 °C. Then, liquid carbon dioxide systematically replaced the acetone through repeated flushings, facilitating the gradual replacement of acetone with liquid carbon dioxide. Subsequently, the temperature was gradually increased above carbon dioxide's critical point to 40 °C, while the pressure was maintained below 90 bar. Lastly, all the carbon dioxide was gradually vented from the chamber. The complete drying procedure took ≈60 min in total.

### Photoresist Composition

In this study, the photoresist was formulated with the following mass fractions: 14% PETMP, 11% DVE‐3, 2% TPO, 0.01% TEMPO, and 75% PEG 200. The constituents were combined in an amber glass container to protect light‐sensitive components and subjected to ultrasonic bath treatment for 30 min, resulting in a homogeneous mixture. After preparation, the mixture was refrigerated overnight and utilized within 48 h for optimal performance.

### Fabrication of Fluorinated and Lubricated Porous Micropillars

Within porous media, the molecular diffusion processes of water and lubricants within exhibit competitive interactions. To mitigate these interactions, the porous microstructure was first pre‐treated with fluorination. This fluorination treatment adheres to the method outlined in previously published literature.^[^ ^8^ ^]^ For the lubrication step, the fluorinated porous microstructure was immersed in a lubricant. Any excess lubricant was subsequently removed by spin‐coating at 7500 rpm for 40 s, ensuring a uniform coating.

### Impregnation of Functional Liquids into Porous Microstructures

A needle, dipped in a functional liquid such as ionic or fluorescent liquids, was positioned adjacent to the samples. When the porous microstructure was brought into contact with the needle's tip, capillary action spontaneously drew the functional liquid into the microstructure. The impregnation process was halted by withdrawing the needle tip from the microstructure, thus allowing for controlled local impregnation.

### Electrochemical Characterization

The bridge‐like microstructures on the interdigitated electrode were characterized using a PGSTAT302N potentiostat with FRA32M module (Metrohm AG, Switzerland). Cyclic voltammetry was performed across a potential range from −1.0 to 1.0 V at a scan rate of 100 mV s^−1^. EIS was conducted using a two‐electrode setup with an applied potential of 0 V, an excitation amplitude of 100 mV rms, and a frequency range from 1 MHz–100 mHz. The analysis was performed using Nova 2.1.7 software (Metrohm AG, Switzerland). The conductivity of the bridge was calculated based on the equation σ = l/(AR), where σ is the ionic conductivity, R is the resistance of the microstructure obtained from EIS measurements, and A and l are the cross‐sectional area and the length of the porous bridge‐like microstructures, respectively.

## Conflict of Interest

The authors declare no conflict of interest.

## Supporting information



Supporting Information

Supplemental Video 1

Supplemental Video 2

## Data Availability

Research data are not shared.
