# Supplementary material for: Fabrication of 3D Functional Nanocomposites Through Post‐Doping of Two‐Photon Microprinted Nanoporous Architectures
Source: Small. 2024 Dec 17;21(5):2403405. doi: 10.1002/smll.202403405 (PMC11798355; doi:10.1002/smll.202403405)
Supplement: Supplementary file 1 — Supporting Information [file SMLL-21-2403405-s001.docx]

Supporting Information

**Fabrication of 3D Functional Nanocomposites through Post-Doping of Two-Photon Microprinted Nanoporous Architectures**

Junning Zhang, Sida Liu, Kannasoot Kanokkanchana, Mariia Kuzina, Meijun Zhou, Xin Du, Zhongze Gu*, Zheqin Dong*, Pavel A. Levkin*

**Comparison of Pre-Doping, Post-Doping, and Other Post-Functionalization Methods**

The main advantages of the pre-doping method are that it offers stable and uniform functional materials and enables one-step fabrication. However, its primary drawbacks include optical irregularities in hybrid resins that can interfere with photopolymerization (*Small* **2019**, 15, 1902687), poor dispersibility of functional dopants, which limits the loading capacity (*Adv. Funct. Mater.* **2023**, 33, 2211280), and compatibility issues between the dopants and the resin, restricting the selection of functional materials (*Adv. Funct. Mater.* **2023**, 33, 2214211). Additionally, a significant amount of time and effort is required to fine-tune the composition and optimize processing parameters.

In contrast, the post-doping method provides greater flexibility in the selection of functional dopants, making it a more versatile approach. It also allows for the rapid preparation of functional microstructures by simply infusing them into porous microstructures. However, the limitations of this method include the fact that the system remains heterogeneous, functional components may leach out or deliquesce, and it requires specific environmental conditions to maintain stability, depending on the dopant used.

Other post-fabrication techniques, such as surface chemical modification or physical deposition, share similarities with the post-doping method but are limited to surface modification (*Adv. Sci.* **2023**, 10, 2204072). The post-doping method can be seen as an extension of these techniques from two-dimensional to three-dimensional applications. Moreover, these methods are limited to grafting chemical groups or depositing a narrow selection of metals or semiconductor materials, which still presents limitations.

In summary, both pre-doping and post-doping methods have their respective advantages and limitations and should be considered complementary approaches rather than replacements for one another.


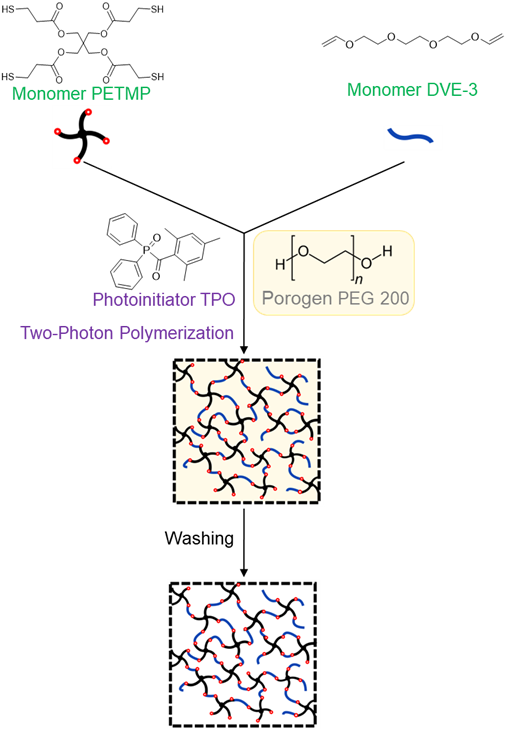


Figure S1. Schematic of the polymerization process of the porous material. After the polymerization of PETMP and DVE-3, the porogen remains in the polymer network as a non-polymerized phase. Upon removal of the porogen, an interconnected porous polymer network is formed.


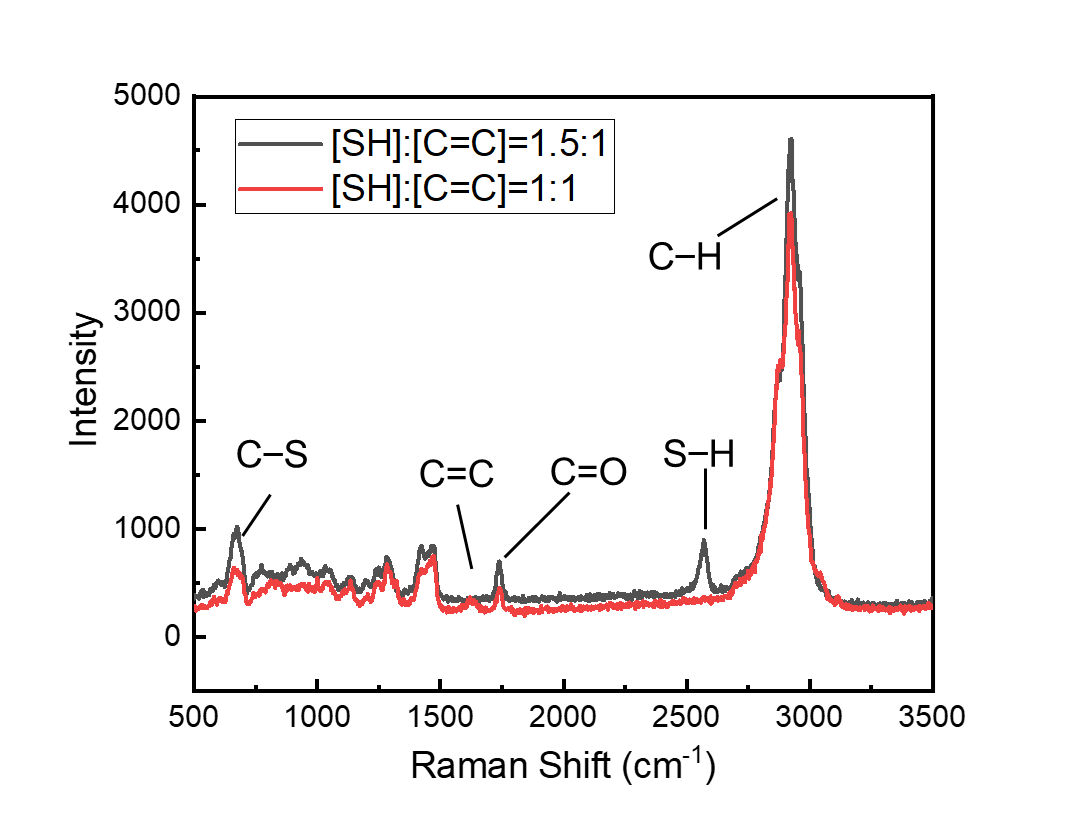


Figure S2. Raman spectra of the two porous materials. By adjusting the ratio of PETMP to DVE-3, the molar ratios of [SH] to [C=C] in the materials were set to 1.5:1 and 1:1, respectively.


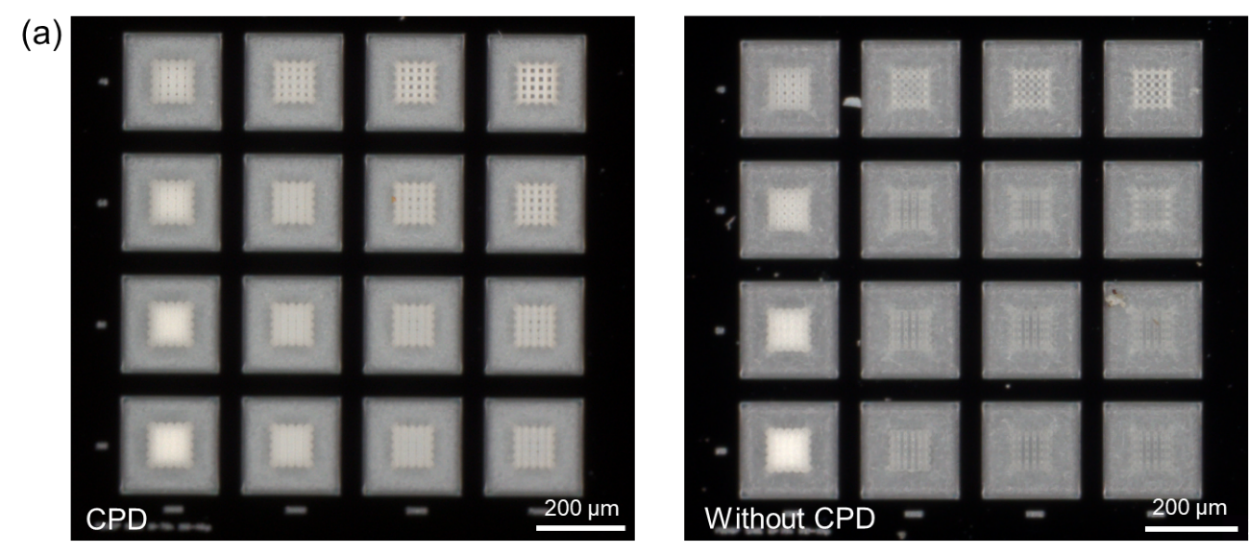


Figure S3. Woodpile porous microstructure obtained with or without critical point drying.


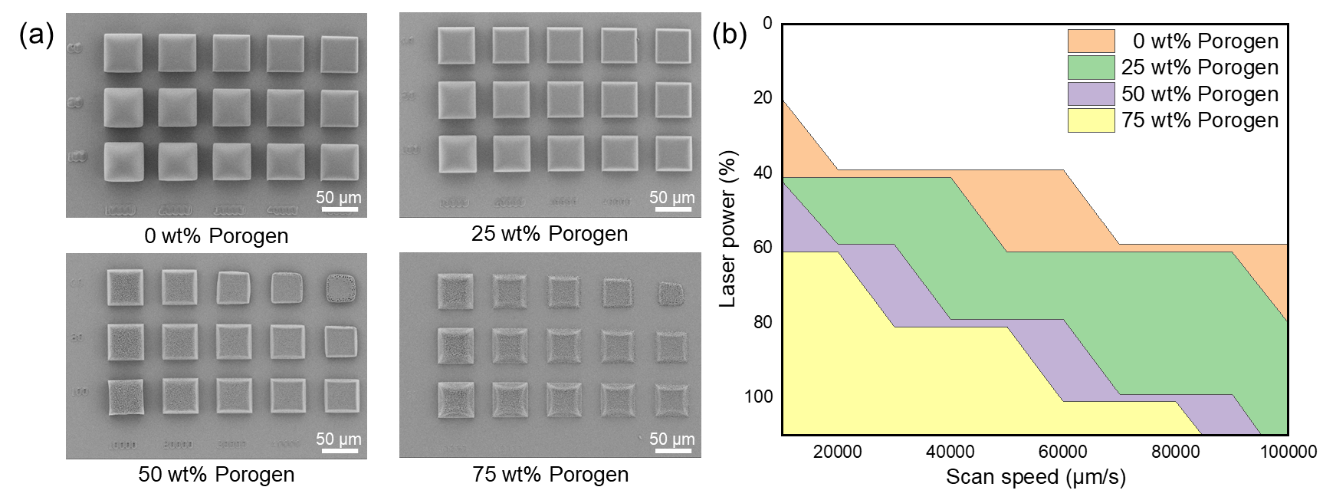


Figure S4. a) SEM images of TPL porous microstructures with increasing porogen concentrations at 0 wt%, 25 wt%, 50 wt%, and 75 wt%. b) Printing diagram for optimized resolution as a function of laser power and scan speed.


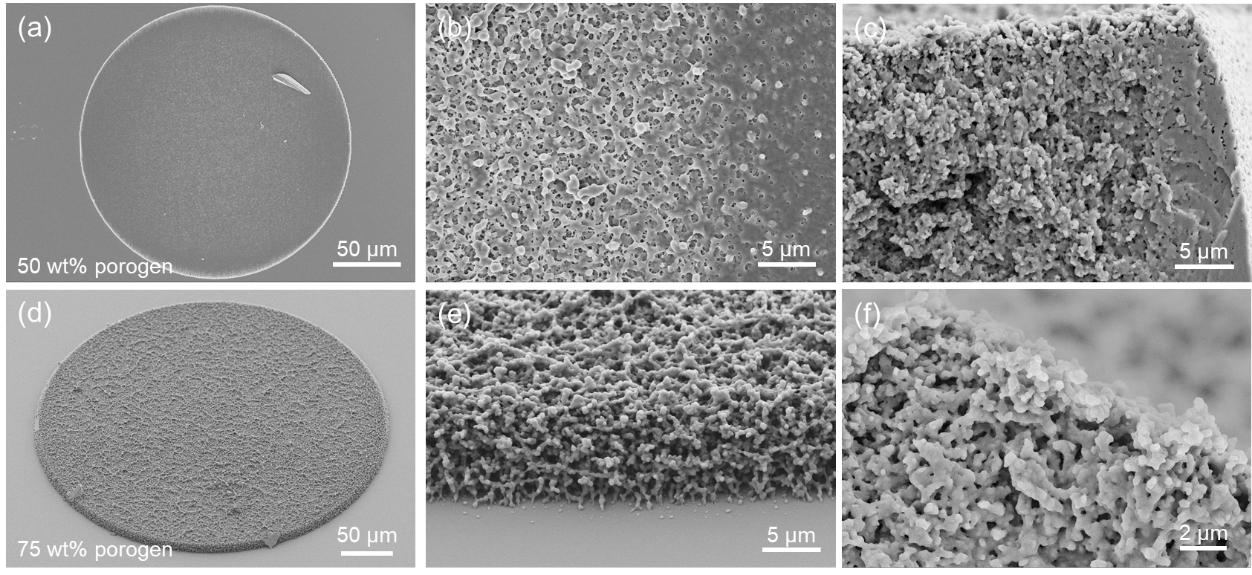


Figure S5. SEM images showing the effect of porogen concentration on pore distribution. (a-c) Top view, local top view, and cross-sectional view of the porous microstructure with a porogen concentration of 50 wt%. (d-f) Top view, local top view, and cross-sectional view of the porous microstructure with a porogen concentration of 75 wt%.


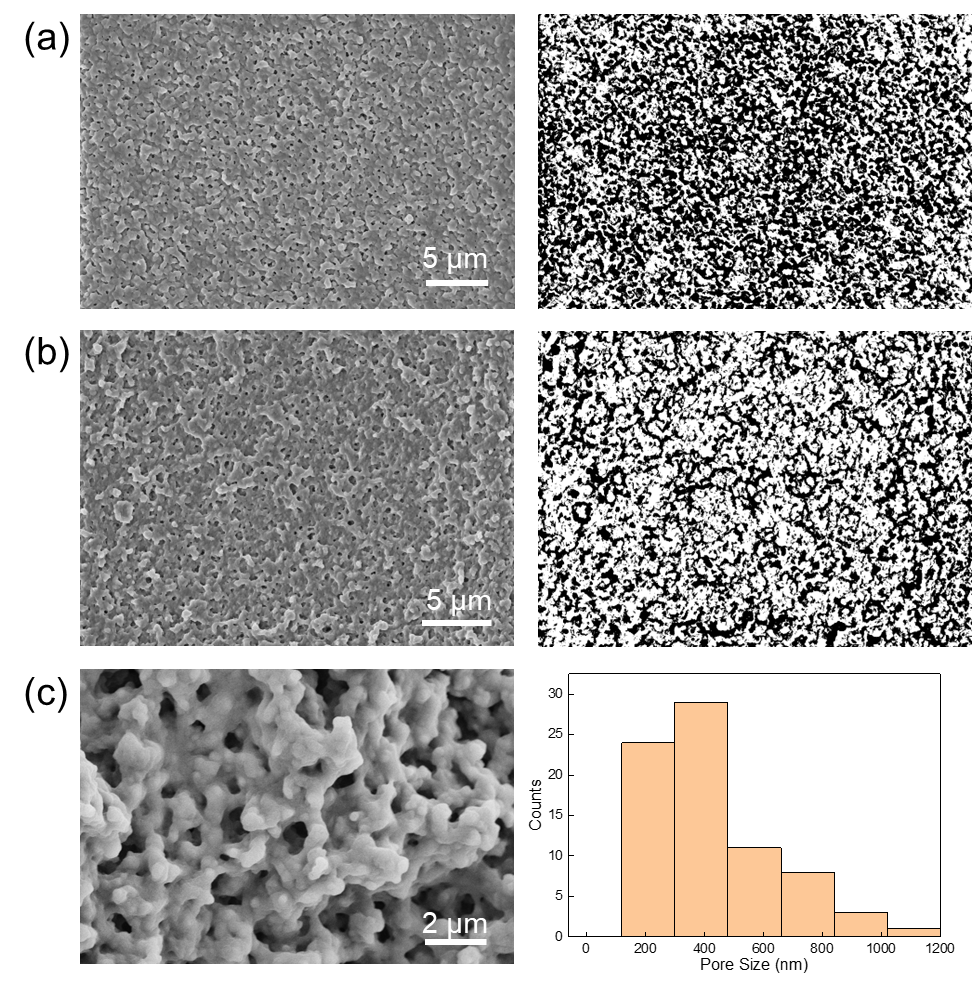


Figure S6. Porosity and pore size analysis of porous microstructures using ImageJ. For porosity analysis, SEM images were first converted to 8-bit mode. Automatic thresholding was then applied to segment the images into binary format, where white and black areas represent pores and the polymer, respectively. For pore size distribution analysis, pores were initially marked manually. Subsequently, their sizes were estimated with ImageJ. (a-b) SEM and corresponding binary images of porous microstructures at 50 wt% and 75 wt% porogen concentrations, respectively. (c) SEM images and pore size distribution for cross-sections of porous microstructures.


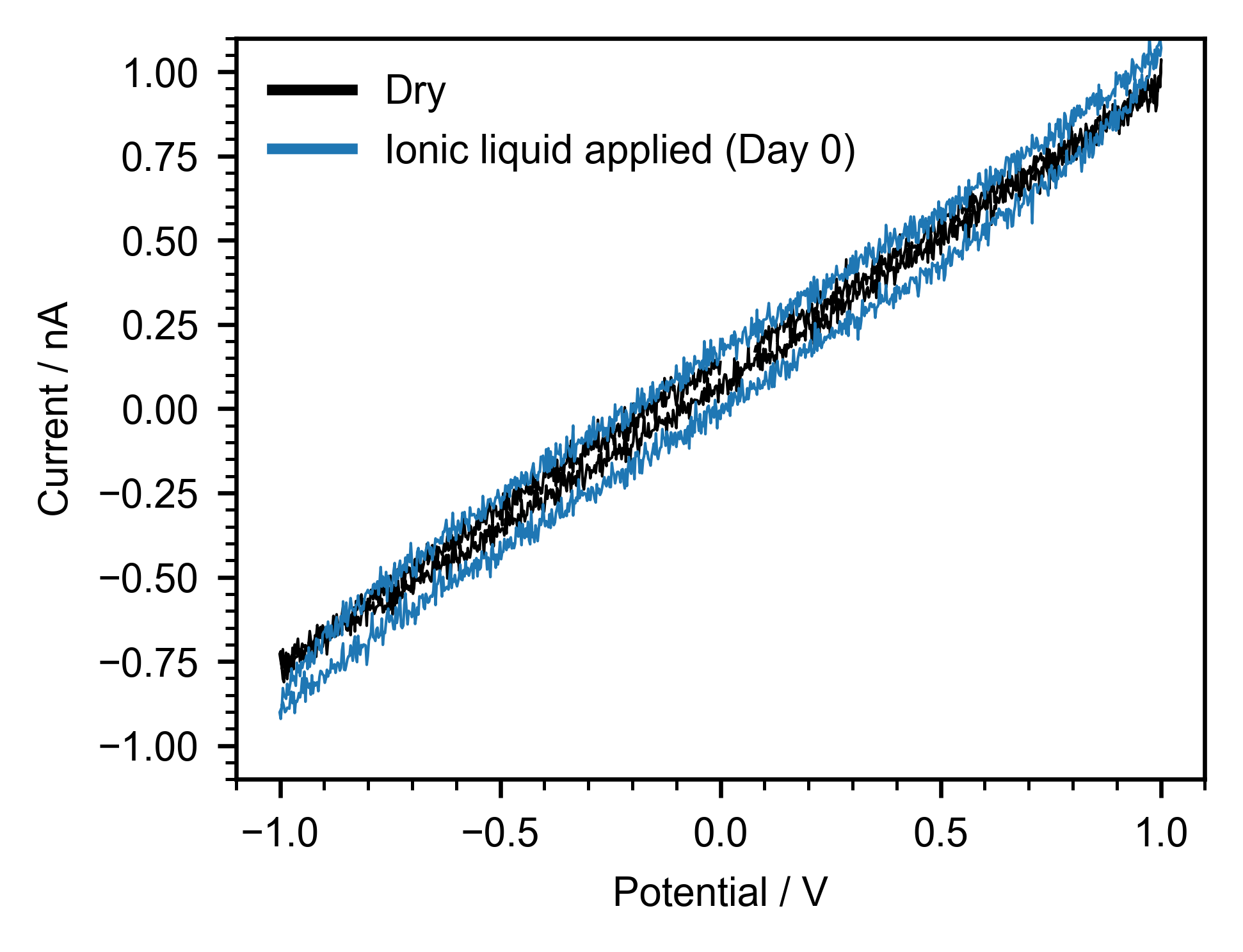


Figure S7. The cyclic voltammogram of the porous microstructures filled with ionic liquids (Day 0).


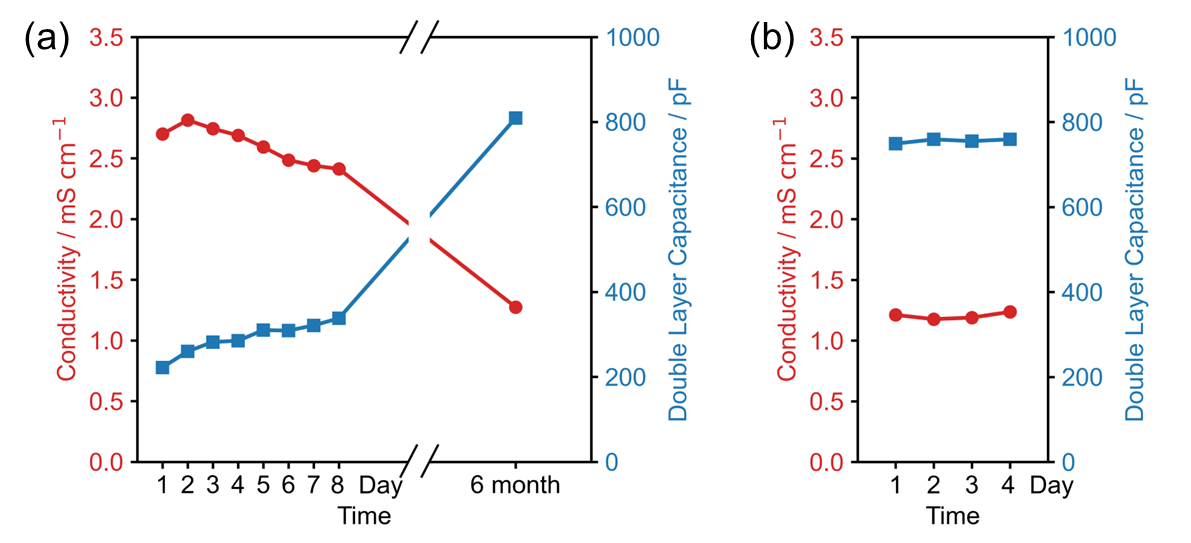


Figure S8 a) Conductivity and double-layer capacitance of the bridge infused with fresh, moisture-free ionic liquid, measured daily from the 1st to the 8th day, and again after 6 months. b) Conductivity and double-layer capacitance of a newly prepared bridge sample infused with the 6-month-old ionic liquid, stored under ambient conditions, measured daily from the 1st to the 4th day. The fact that the newly infused sample (with 6-month-old, similar batch of ionic liquid) resembles the conductivity and the capacitance of the 6-month-old original sample suggests that the decrease of conductivity is primarily due to the degradation of the ionic liquid itself, not the infused bridge structure.


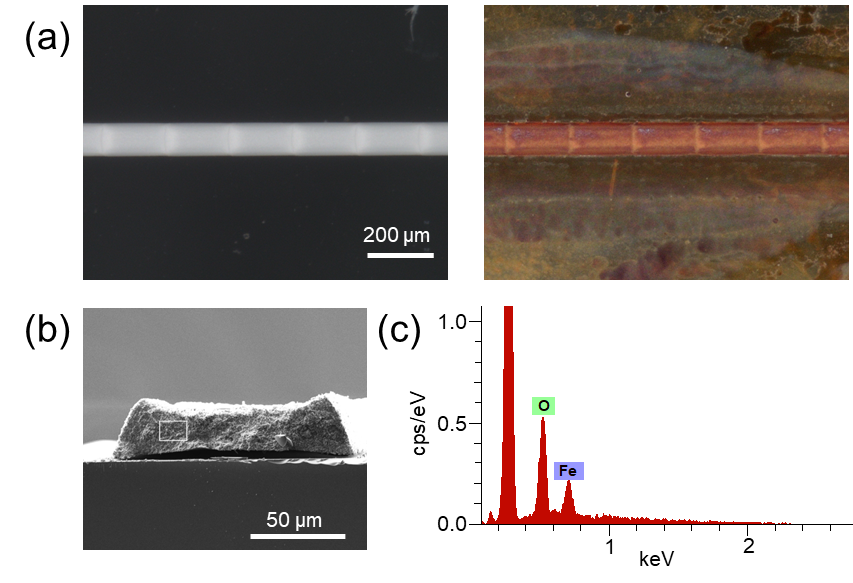


Figure S9. a) Optical images of porous microstructures showing before and after the adsorption of Fe_3_O_4_ nanoparticles. SEM (b) and EDX (c) characterization of a cross-sectional view of a porous microstructure with Fe_3_O_4_ nanoparticles adsorbed.


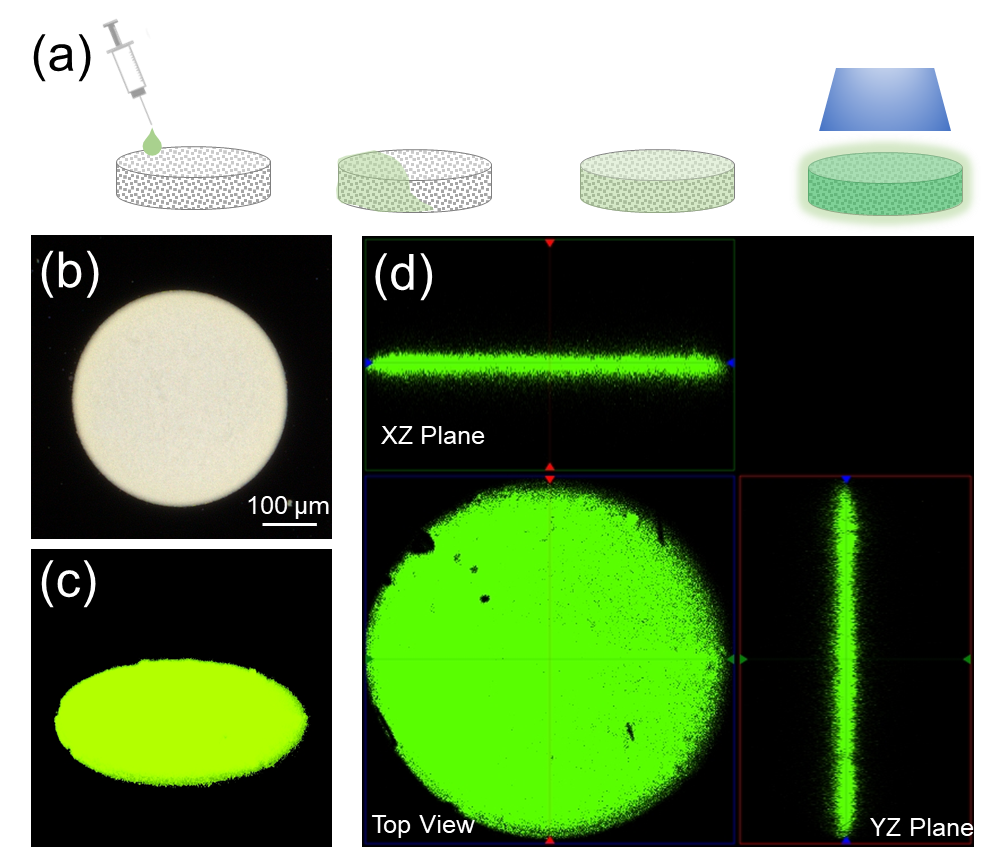


Figure S10. a) Schematic of fluorescent liquid infusion into the porous microstructure and observation using confocal microscopy. b) Original optical image of the porous microstructure. c) Confocal image of the porous microstructure filled with fluorescent liquid, including a 3D reconstruction with z-stack planes. d) Sectional view of the microstructure in the XZ- and YZ-planes.
